# Combining Protein and Strain Engineering for the Production of Glyco-Engineered Horseradish Peroxidase C1A in *Pichia pastoris*

**DOI:** 10.3390/ijms161023127

**Published:** 2015-09-24

**Authors:** Simona Capone, Lejla Ćorajević, Günther Bonifert, Patrick Murth, Daniel Maresch, Friedrich Altmann, Christoph Herwig, Oliver Spadiut

**Affiliations:** 1 Institute of Chemical Engineering, Research Area Biochemical Engineering, Vienna University of Technology, Gumpendorfer Strasse 1a, 1060 Vienna, Austria; E-Mails: simona.capone@tuwien.ac.at (S.C.); lejla.corajevic91@gmail.com (L.C.); guenther.bonifert@chello.at (G.B.); patrick.murth@gmx.at (P.M.); christoph.herwig@tuwien.ac.at (C.H.); 2 Department of Chemistry, University of Natural Resources and Life Sciences, 1180 Vienna, Austria; E-Mails: maresch@gmx.at (D.M.); friedrich.altmann@boku.ac.at (F.A.)

**Keywords:** horseradish peroxidase, glyco-engineering, strain engineering, bioreactor cultivation, OCH1, site-directed mutagenesis

## Abstract

Horseradish peroxidase (HRP), conjugated to antibodies and lectins, is widely used in medical diagnostics. Since recombinant production of the enzyme is difficult, HRP isolated from plant is used for these applications. Production in the yeast *Pichia pastoris* (*P. pastoris*), the most promising recombinant production platform to date, causes hyperglycosylation of HRP, which in turn complicates conjugation to antibodies and lectins. In this study we combined protein and strain engineering to obtain an active and stable HRP variant with reduced surface glycosylation. We combined four mutations, each being beneficial for either catalytic activity or thermal stability, and expressed this enzyme variant as well as the unmutated wildtype enzyme in both a *P. pastoris* benchmark strain and a strain where the native α-1,6-mannosyltransferase (OCH1) was knocked out. Considering productivity in the bioreactor as well as enzyme activity and thermal stability, the mutated HRP variant produced in the *P. pastoris* benchmark strain turned out to be interesting for medical diagnostics. This variant shows considerable catalytic activity and thermal stability and is less glycosylated, which might allow more controlled and efficient conjugation to antibodies and lectins.

## 1. Introduction

The methylotrophic yeast *Pichia pastoris* is a widely used host organism for recombinant protein production. It can grow on cheap and defined media to high cell densities, is robust against stressful conditions and able to perform post-translational modifications, like glycosylation [1,2,3,4]. However, glycosylation in *Pichia pastoris* (*P. pastoris*) is characterized by a severe drawback: native glycosyltransferases recognize the amino acid motif Asn-X-Ser/Thr and link many glycan moieties to the asparagine residues of recombinant proteins yielding a hyperglycosylated product (e.g., [5,6,7]). Hyperglycosylation causes many problems as it alters the physicochemical properties of the product, hampers downstream processing [5,8] and prevents medical applications. One of the main disadvantages of yeast-derived glycosylation lies in its heterogeneous nature making subsequent conjugation to antibodies and lectins, a prerequisite for applications in medical diagnostics, extremely difficult. Two different approaches can be applied to reduce yeast-derived hyperglycosylation, namely (1) protein engineering and (2) strain engineering. The yeast *P. pastoris* is a prominent example for such efforts. In several studies detailed strain engineering approaches describing humanization of this yeast were reported (Table 1; e.g., [7,8,9,10,11,12,13,14]).

**Table 1:** Selected studies focusing on the humanization of *N*-glycosylation in *P. pastoris*.

Goal of the Study	Citation
Introduction of α-1,2-Mns and GntI, *OCH1* inactivation via a knock-in plasmid	[9]
Introduction of an UDP-GlcNAc transporter, α-1,2-MnsIA, MnsII, GntI, GntII in a *Δoch1*::*URA3* strain	[10]
Introduction of sialic acid biosynthesis pathway to produce sialylated glycoproteins	[11]
*OCH1* knock-out and introduction of glycosidases and glycosyltransferases to produce terminally galactosylated glycoproteins	[14]

Mns, mannosidase; Gnt, β-*N*-acetylglucosaminyltransferase; UDP-GlcNAc, uridine diphosphate-*N*-acetylglucosamine; *OCH1*, outer chain elongation gene.

We used *P. pastoris* for the recombinant production of the heme-containing plant enzyme horseradish peroxidase (HRP), an enzyme widely used in medical diagnostics (e.g., [5,15]). We chose this yeast as expression host because Morawski *et al.* [16,17] had shown *P. pastoris* to produce recombinant proteins with significant shorter surface glycans than *Saccharomyces cerevisiae* (*S. cerevisiae*). We investigated various aspects of the expression of HRP in *P. pastoris*: different HRP isoenzymes were produced [18,19], production strategies were developed and optimized [20,21,22,23], media supplementation and strain engineering for increased heme-incorporation were analyzed [24] and the methanol utilization pathway of *P. pastoris* was manipulated for higher productivity [25].

In more recent studies we applied both protein and strain engineering with the goal of producing more homogenously glycosylated HRP variants in *P. pastoris*. However, we did not use fully humanized yeast strains, which are proprietary, but produced HRP in a *P. pastoris* strain with a deleted *och1* gene (Δoch1 strain; [26]). This gene codes for an α-1,6-mannosyltransferase responsible for triggering the uncontrolled addition of mannose residues to the recombinant protein. Although HRP produced in the Δoch1 strain was more homogeneously glycosylated with more than 70% of the enzyme being of the Man_8_ glycosylation type, the Δoch1 strain showed a growth impaired phenotype and was hard to cultivate [26]. In a subsequent study, we analyzed the effect of the process parameters temperature, pH and dissolved oxygen concentration on strain physiology and productivity [6]. We found that the space-time-yield (STY) of the glyco-engineered strain was eight-fold lower compared to an unmodified *P. pastoris* benchmark strain, making HRP production in the Δoch1 strain unattractive.

Thus, we glyco-engineered the enzyme HRP by mutating each of the eight *N*-glycosylation sites to produce in a benchmark strain but still get HRP with reduced surface glycosylation [27]. We found different effects of the single mutations on enzyme activity and stability and demonstrated that mutation of the respective *N*-glycosylation site in fact resulted in the absence of glycans there [27]. Although the combination of all eight mutations gave a non-glycosylated enzyme, both catalytic activity and thermal stability were dramatically reduced leading to the hypothesis that some of the *N*-glycosylation sites must not be mutated to obtain active and stable HRP.

In the present study we combined both (1) protein engineering and (2) strain engineering to obtain a stable and active HRP variant with reduced and more homogeneous surface glycosylation. We combined the four mutations we had identified as being beneficial for either catalytic activity or stability in a previous study (Table 2; [27]).

**Table 2:** Biochemical characteristics of the unmodified horseradish peroxidase (HRP) wildtype enzyme (wt) and four different HRP variants [27].

Enzyme	ABTS			H_2_O_2_			Thermal Half-Life Time
Variant	*K*_m_ (mM)	*v*_max_ (U/mg)	*v*_max_/*K*_m_ (U/mg/mM)	*K*_m_ (mM)	*v*_max_ (U/mg)	*v*_max_/*K*_m_ (U/mg/mM)	τ_½_ (min)
wt	1.60	44.2	27.7	0.003	16.3	5433	20.6
N13D	2.90	47.2	16.3	0.005	14.7	3066	28.9
N57S	2.98	113	38.1	0.004	23.7	5378	38.5
N255D	1.72	51.5	29.9	0.005	21.6	4506	11.6
N268D	1.89	32.5	17.2	0.003	10.6	3642	61.9

ABTS, 2-2-azinobis-(3-ethylbenzothiazoline-6-sulfonic acid); H_2_O_2_, hydrogen peroxide.

We produced the enzyme variant, called 4/8 HRP, in both an unmodified *P. pastoris* benchmark strain and a Δoch1 strain. Summarizing, enzymes produced in the Δoch1 strain were characterized by lower catalytic activity and stability. However, 4/8 HRP produced in the *P. pastoris* benchmark strain (enzyme wt^4/8 HRP^) showed considerable catalytic activity and increased thermal stability. In combination with its significantly reduced surface glycosylation, which might allow more controlled conjugation to antibodies and lectins, this variant might be useful for applications in medical diagnostics in the future.

## 2. Results and Discussion

### 2.1. Strain Physiology

**Figure 1:**
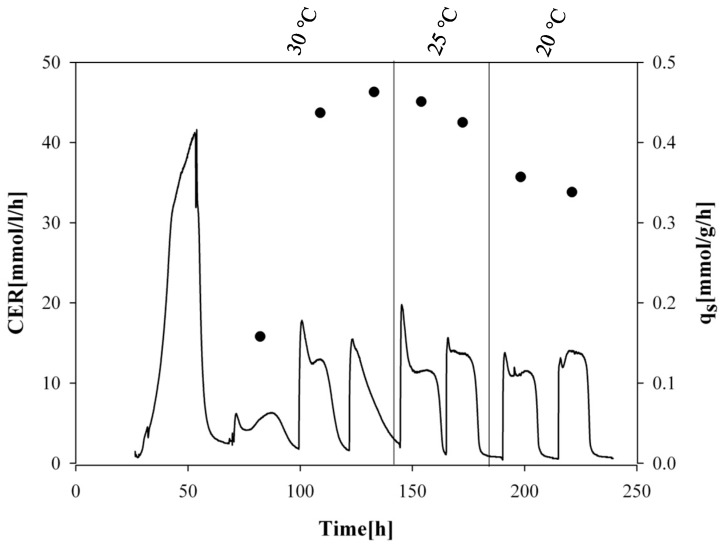
Schematic overview of the dynamic batch cultivation of strain wt^4/8 HRP^ with methanol pulses at different temperatures. Black continuous line, carbon dioxide evolution rate (CER); black dots, specific methanol uptake rate (q_s MeOH_).

We cultivated the four recombinant *P. pastoris* strains in dynamic batch cultivations with methanol pulses at different temperatures and analyzed the effect of temperature on strain physiology. A typical cultivation is exemplarily shown for strain wt^4/8 HRP^ in Figure 1.

The most important strain physiological parameters are summarized in Table 3. Closing C-balances for the benchmark strains underline data validity. Similar to our previous studies, we observed that the Δoch1 strains lost metabolic activity over time and were affected by cell lysis [26]. Thus, C-balances did not close.

When comparing the recombinant benchmark strains, great differences in specific methanol uptake rates (q_s MeOH_) were identified. While for both strains q_s MeOH_ increased with increasing temperature, a correlation we also had observed before [6], the strain producing the 4/8 HRP variant showed a three-fold lower q_s MeOH_ compared to the strain producing the unmutated enzyme at the respective temperature. Apparently, production of the glyco-engineered 4/8 HRP caused a physiological burden for the yeast, decelerating metabolism and thus methanol uptake.

When comparing the recombinant Δoch1 strains, we observed that q_s MeOH_ for both strains decreased with increasing temperature, a phenomenon we also had described before [6]. However, q_s MeOH_ of both Δoch1 strains were comparable at each temperature indicating that the mutated product did not cause any physiological burden. We speculate that the glyco-engineered 4/8 HRP variant might cause problems in the *P. pastoris* benchmark strain during secretion as it might get stuck in the cell wall. On the contrary, the same enzyme variant can be secreted without any problems in the Δoch1 strain, which has a completely altered cell wall structure [24]. However, this remains to be elucidated in detail.

**Table 3:** Physiological parameters of strains wt^wt HRP^, benchmark strain expressing the unmutated HRP enzyme; wt^4/8 HRP^, benchmark strain expressing the mutated 4/8 HRP variant; OCH1^wt HRP^, deleted OCH1 gene strain expressing the unmutated HRP enzyme and OCH1^4/8 HRP^ delete OCH1 gene strain expressing the mutated 4/8 HRP variant were determined in dynamic batch cultivations.

Strain	μ_max gly_ (h^−1^)	Δ_time adapt_ (h)	q_s adapt_ (mmol/g/h)	q_s MeOH 20 °C_ (mmol/g/h)	q_s MeOH 25 °C_ (mmol/g/h)	q_s MeOH 30 °C_ (mmol/g/h)	C-Balance
wt^wt HRP^	0.271	11.1	0.272	0.931	1.190	1.32	0.96
wt^4/8 HRP^	0.200	16.0	0.158	0.354	0.438	0.450	0.97
OCH1^wt HRP^	0.199	4.5	0.370	0.891	0.780	0.632	const. Decreasing
OCH1^4/8 HRP^	0.182	3.8	0.400	1.02	0.955	0.800	const. Decreasing

### 2.2. Protein Purification

After harvest, we purified the different enzyme variants by a 1-step hydrophobic charge interaction chromatography (HCIC) purification strategy [8,19]. As shown in Table 4, the majority of each HRP variant was found in the respective flow-through fraction, where purification factors (PF) between 1.34 and 1.53 were achieved. However, enzyme OCH1^4/8 HRP^ showed a different result, as nearly 20% of the enzyme was retained on the resin. The eluate fraction showed a higher PF compared to the flow-through fraction (Table 4). We ascribe this phenomenon to the fact that this HRP variant was the least glycosylated one, as the four remaining glycosylation sites mainly carried Man_8_ instead of longer Man chains (respective detailed analyses had been performed in our previous study [26]). Apparently, the reduced glycosylation of enzyme OCH1^4/8 HRP^ allows physico-chemical interactions of the enzyme variant and the resin. One might speculate that the amount of enzyme variant able to interact with the resin should be even higher. We think that the rather stressful manner of cultivation with pulses and temperature shifts might have caused very heterogeneous glycosylation on the four remaining *N*-glycosylation sites and thus resulted in the still rather low fraction of only around 20% enzyme interacting with the resin. Detailed analysis of surface glycosylation by mass spectrometry, as we have done previously [24], could shed light on this speculation. However, we did not perform this analysis in this study, since variant OCH1^4/8 HRP^ did not turn out to be interesting for further applications due to low catalytic activity (see below).

**Figure 2:**
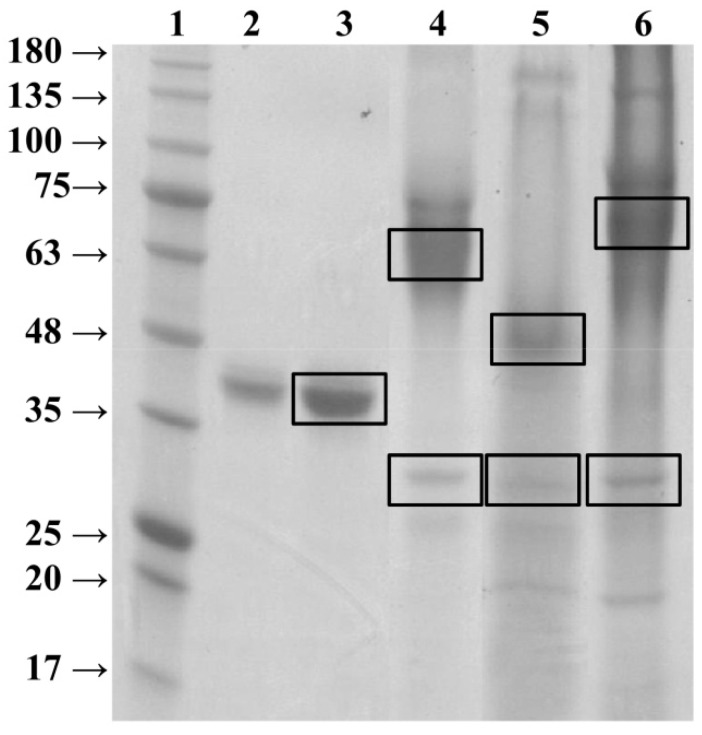
Sodium dodecyl sulfate polyacrylamide gel electrophoresis (SDS-PAGE) of different horseradish peroxidase (HRP) variants. Black boxes indicate protein bands analyzed by mass spectrometry. Lane 1, BLUeye Prestained Protein Ladder; lanes 2 and 3, HRP from plant in two different concentrations (4 μg protein and 8 μg protein, respectively); lane 4, wt^wt HRP^, unmutated HRP enzyme expressed in the benchmark strain; lane 5, wt^4/8 HRP^, mutated 4/8 HRP variant expressed in the benchmark strain; lane 6, OCH1^4/8 HRP^, mutated 4/8 HRP variant expressed in the deleted OCH1 gene strain.

After HCIC, flow-through fractions showing highest PFs were pooled and concentrated to around 1.5 mL. To assess purity of the different enzyme preparations Reinheitszahl values (RZ; A_404_/A_280_) were determined (Table 4). Highly pure HRP preparations are known to have RZ values of more than 3.0 [16]. Although we did not get pure enzyme preparations by the one-step HCIC purification in this study, the RZ values of the different enzyme variants were in the same range. We also analyzed the different enzyme variants on SDS-PAGE gels, to identify potential differences in apparent size (Figure 2). Furthermore, interesting protein bands were excised and analyzed by mass spectrometry (respective bands are indicated in black boxes in Figure 2).

**Table 4:** Results of the hydrophobic charged interaction chromatography (HCIC) purification and Reinheitszahl (RZ) measurements for the four different HRP enzyme variants.

HCIC					Concentrated Fraction
Enzyme Variant	R% Total	R% FT	PF FT	Specific Activity (U/mg)	RZ (A_404_/A_280_)
wt^wt HRP^	87.2	87.2	1.49	273.3	0.50
wt^4/8 HRP^	97.4	95.6	1.34	36.4	0.51
OCH1^wt HRP^	99.8	98.7	1.53	59.8	0.63
OCH1^4/8 HRP^	93.1	76.1	1.47	11.5	0.19
**Variant**	**R% Total**	**R% Eluate**	**PF Eluate**	**Specific Activity (U/mg)**	**RZ (A_404_/A_280_)**
OCH1^4/8 HRP^	93.1	17.5	2.43	19.1	0.31

The enzyme preparation from plant showed a rather distinct band at an apparent size of around 40 kDa on the SDS gel (lanes 2 and 3 in Figure 2), whereas the recombinant enzyme wt^wt HRP^ showed a smear at an apparent size of around 65 kDa (lane 4 in Figure 2). Both proteins were identified as HRP by mass spectrometry (Table 5). The apparent size difference of around 20 kDa and the smeary appearance of wt^wt HRP^ result from the heterogeneous yeast-derived glycosylation [17,20,21]. The second prominent protein band in lane 4 at an apparent size of around 30 kDa was identified as a glucosidase from *P. pastoris* (Table 5).

As shown in Figure 2, the preparation of enzyme wt^4/8 HRP^ showed a different protein pattern on the SDS gel, as the band at an apparent size of 65 kDa disappeared, whereas a prominent band at an apparent size of around 50 kDa appeared. In fact, this protein was identified as HRP (Table 5). Apparently, the mutation of four of the eight *N*-glycosylation sites resulted in the absence of glycans there and thus a size reduction of around 15 kDa. This nicely underlines the feasibility of reducing the vast and heterogeneous glycosylation of recombinant proteins from yeast by protein design. The second prominent band at an apparent size of around 30 kDa was again identified as a glucosidase (Table 5).

Lane 6 in Figure 2 shows the protein bands of enzyme preparation OCH1^4/8 HRP^. Again, we observed a different protein pattern. The prominent band at an apparent size of around 70 kDa was identified as an oxidase and the band at an apparent size of around 30 kDa again as a glucosidase (Table 5). We expected to see a protein band at an apparent size of around 45 kDa representing enzyme OCH1^4/8 HRP^. However, no respective band was visible on the SDS gel. We ascribe this absence to the extremely low protein production in the Δoch1 strain [6,26]. Furthermore, cell lysis during bioreactor cultivation resulted in a rather high impurity pattern, which is also demonstrated by the low RZ value for this enzyme variant (Table 4). However, we still measured enzymatic activity for OCH1^4/8 HRP^ and thus included this enzyme variant in the comparative biochemical characterization.

**Table 5:** Identification of prominent protein bands by mass spectrometry.

Lane	Apparent Size [kDa]	Rank	Peptides	Scores	Protein	Accession
3	45	1	11	608.9	Peroxidase C1A Organism species (OS)=Armoracia rusticana	PER1A_ARMRU
4	65	1	6	386.9	1,3-β-glucanosyltransferase OS=Komagataella pastoris	Q0QCW1_PICPA
		2	9	368.0	Peroxidase C1A OS=Armoracia rusticana	PER1A_ARMRU
	30	1	9	411.5	Glucan 1,3-β-glucosidase OS=Komagataella pastoris	F2QPL8_PICP7
5	50	1	9	454.5	Alpha-1-antichymotrypsin 2 OS=Sus scrofa	Q9GMA6_PIG
		2	9	386.6	Keratin, type II cytoskeletal 1 OS=Homo sapiens	K2C1_HUMAN
		3	9	317.4	Peroxidase C1A OS=Armoracia rusticana	PER1A_ARMRU
	30	1	9	411.5	Glucan 1,3-β-glucosidase OS=Komagataella pastoris	F2QPL8_PICP7
6	70	1	20	1136.6	Primary-amine oxidase OS=Komagataella pastoris	F2QTE6_PICP7
		2	10	685.6	1,3-β-glucanosyltransferase OS=Komagataella pastoris	Q0QCW1_PICPA
		3	9	548.8	1,3-β-glucanosyltransferase OS=Komagataella pastoris	F2QQJ2_PICP7
	30	1	9	411.5	Glucan 1,3-β-glucosidase OS=Komagataella pastoris	F2QPL8_PICP7

### 2.3. Biochemical Enzyme Characterisation

After purification, we biochemically characterized the different HRP variants. The kinetic parameters for ABTS and H_2_O_2_ are summarized in Table 6. We also included plant HRP for comparison. Introducing the four mutations N13D, N57S, N255D and N268D into HRP did not affect the affinity towards ABTS and H_2_O_2_ as *K*_m_ values of enzymes wt^wt HRP^ and wt^4/8 HRP^ were comparable. However, the catalytic efficiency was reduced seven-fold for ABTS and six-fold for H_2_O_2_, respectively. Apparently, the introduced mutations did not only reduce surface glycosylation (Figure 2) but also affected the active site and reduced catalytic activity.

When we produced the HRP variants in the Δoch1 strain, we found that reduced surface glycosylation, caused by the absence of the native enzyme OCH1 [26], did not alter substrate affinity either. In fact, *K*_m_ values of all four HRP variants for both ABTS and H_2_O_2_ were comparable. However, reducing surface glycosylation affected catalytic efficiency. Enzyme OCH1^wt HRP^ showed a nearly six-fold reduced *v*_max_/*K*_m_ compared to enzyme wt^wt HRP^. Apparently, the benefit of having a more homogeneously glycosylated enzyme produced in the Δoch1 strain comes at cost of catalytic activity. Finally, we characterized the mutated 4/8 HRP produced in the Δoch1 strain (enzyme OCH1^4/8 HRP^). This enzyme is the least glycosylated one in this study, only providing four out of eight *N*-glycosylation sites, which are mainly occupied by Man_8_-glycan structures [26]. However, compared to enzyme wt^wt HRP^ the catalytic efficiency for ABTS was reduced 119-fold and for H_2_O_2_ 76-fold, respectively. Although, enzyme OCH1^4/8 HRP^ is basically fit for medical applications, as it misses the heterogeneous yeast-derived glycan structures, the highly reduced catalytic activity as well as low productivity in the bioreactor will most likely prevent future applications.

**Table 6:** Kinetic constants of four different HRP enzyme variants and plant HRP.

Enzyme	ABTS			H_2_O_2_		
Variant	*K*_m_ (mM)	*v*_max_ (U/mg)	*v*_max_/*K*_m_ (U/mg/mM)	*K*_m_ (mM)	*v*_max_ (U/mg)	*v*_max_/*K*_m_ (U/mg/mM)
wt^wt HRP^	1.50	152.9	101.9	0.009	55.2	6133
wt^4/8 HRP^	0.99	13.6	13.7	0.015	15.5	1030
OCH1^wt HRP^	1.56	26.8	17.2	0.008	10.4	1300
OCH1^4/8 HRP^	1.34	1.28	0.96	0.016	1.29	80.7
plant HRP	1.75	567.2	324.8	0.033	377.8	11,589

ABTS, 2-2-azinobis-(3-ethylbenzothiazoline-6-sulfonic acid); H_2_O_2_, hydrogen peroxide.

Compared to the commercially available HRP preparation from plant, all recombinant HRP variants showed comparable *K*_m_ values but reduced catalytic activity. This might be a result from incomplete heme incorporation or different surface glycosylation. However, the plant preparation contains a mixture of different HRP isoenzymes with varying surface glycosylation, which has to be isolated from its natural source in a rather cumbersome way. Furthermore, seasonal variation in the isoenzyme content and thus the variable production scenario describe only some of the disadvantages of plant HRP. Thus, even though recombinant HRP variants show lower catalytic activity, they are still interesting for industry, as they only describe a single isoenzyme that can be produced in a predictable manner in the controlled environment of a bioreactor. Furthermore, less glycosylated enzymes might allow more controlled and efficient conjugation to antibodies and lectins, which outweighs reduced catalytic activity.

### 2.4. Thermal Stability

Since we had determined mutations N13D, N57S and N268D to positively affect thermal stability of HRP before (Table 2; [27]), we also analyzed thermal half-life times of the four HRP variants at 60 °C. We again included plant HRP as standard and normalized all enzyme preparations to a protein concentration of 0.1 mg/mL before incubation to guarantee comparability. In Table 7 the respective results are summarized.

**Table 7:** Thermal half-life times of four different HRP variants and the HRP plant preparation at 60 °C. All enzymes were normalized to a protein concentration of 0.1 mg/mL before incubation.

Enzyme Variant	Protein Concentration (mg/mL)	τ_1/2 60 °C_ (min)
wt^wt HRP^	0.1	31.5
wt^4/8 HRP^		173.2
OCH1^wt HRP^		3.3
OCH1^4/8 HRP^		19.3
plant HRP		53.3

As shown in Table 7, the introduction of the four mutations into HRP caused a significant increase in thermal stability. Enzyme wt^4/8 HRP^ had a 5.5-fold higher τ_1/2 60 °C_ than enzyme wt^wt HRP^ and was even 3.3-fold more stable than the enzyme preparation from plant. The same HRP variants produced in the Δoch1 strain showed a 10-fold reduced thermal stability compared to their respective counterparts from the benchmark strain. Reducing glycosylation to Man_8_ structures apparently affected stability to a greater extent than completely removing four *N*-glycosylation sites. In fact, mutating the four *N*-glycosylation sites significantly increased stability instead of decreasing it. It is remarkable that enzyme OCH1^4/8 HRP^ actually showed a similar thermal half-life time as enzyme wt^wt HRP^ (Table 7).

Considering both, kinetic parameters and thermal stability, enzyme wt^4/8 HRP^ might be interesting for future applications in medical diagnostics. This enzyme variant can be efficiently produced in bioreactor cultivations, is less glycosylated, which might allow more controlled and efficient conjugation to antibodies and lectins, still shows considerable catalytic activity and a 5.5-fold higher thermal stability compared to enzyme wt^wt HRP^. In fact, higher stability and reduced glycosylation could compensate for reduced catalytic activity.

## 3. Experimental Section

### 3.1. Chemicals

2,2′-Azino-bis(3-ethylbenzthiazoline-6-sulfonic acid) diammonium salt (ABTS), D(+)-biotin and hemin were purchased from Sigma-Aldrich. Difco™ yeast nitrogen base w/o amino acids (YNB), Bacto™ tryptone and Bacto™ yeast extract were obtained from Becton Dickinson (Franklin Lakes, NJ, USA). Zeocin™ was obtained from InvivoGen (San Diego, CA, USA). Other chemicals were obtained from Carl Roth (Karlsruhe, Germany).

### 3.2. Strain Generation

All strains in this study are based on the *P. pastoris* wildtype strain CBS7435. Generation of the Δoch1 strain and the single HRP variants are described in detail in our previous studies [26,27]. In short, the four Asn, representing glycosylation sites of HRP, were mutated by site directed mutagenesis and subsequent digestion with *Dpn*I. The mutagenic PCR was performed as: 98 °C for 30 s; then 10 cycles of 98 °C for 10 s, 57 °C for 20 s, 72 °C for 1 min-10 cycles of 98 °C for 10 s, 60 °C for 20 s, 72 °C for 1 min-10 cycles of 98 °C for 10 s, 63 °C for 20 s, 72 °C for 1 min; with a final incubation at 72 °C for 10 min. Each reaction contained 1× HF buffer (Fermentas, Waltham, MA, USA), 0.01 μg of plasmid DNA, 2.5 U *Phusion* DNA polymerase (Fermentas), 10 μM of each dNTP and 5 pmol of each primer in a total volume of 50 μL. All primers are listed in Supplementary Table S1 and were purchased from Microsynth (Balgach, Sweden).

After PCR, the methylated template DNA was degraded by digestion with 10 U of *Dpn*I at 37 °C for at least three hours. The remaining PCR products were purified using the QIAquick PCR purification kit (QIAGEN, Hilden, Germany) and 5 μL of each purified PCR product were transformed into electro-competent *E. coli* TOP10 F′ cells. The successful introduction of the desired mutation and the absence of further mutations were confirmed by DNA sequencing (Microsynth). Transformation of approximately 2 μg *Swa*I-linearized pPpT4_S plasmid DNA harbouring the respective mutated HRP gene into the *P. pastoris* benchmark and the Δoch1 strain was done by electroporation. Stable transformants were generated via homologous recombination between the linearized plasmid DNA and genomic yeast DNA. Selection of successfully transformed clones was performed on Yeast Extract Peptone Dextrose medium (YPD; 10 g/L yeast extract, 20 g/L peptone, 20 g/L glucose, 20 g/L agar) supplemented with 100 mg/L Zeocin. In total, we generated and compared four recombinant *P. pastoris* strains producing four different HRP enzyme variants (Table 8).

**Table 8:** Recombinant *P. pastoris* strains and HRP enzyme variants in this study.

*P. pastoris* Chassis Strain	HRP Variant	Name of Recombinant *P. pastoris* Strain and Enzyme
benchmark strain	wt HRP	wt^wt HRP^
	4/8 HRP	wt^4/8 HRP^
Δoch1 strain	wt HRP	OCH1^wt HRP^
	4/8 HRP	OCH1^4/8 HRP^

### 3.3. Bioreactor Cultivations

#### 3.3.1. Preculture

Precultures were grown in 100 mL YNB_Zeo medium (0.1 M potassium phosphate buffer, pH 6.0; 3.4 g/L YNB w/o amino acids and ammonium sulfate, 10 g/L (NH_4_)_2_SO_4_, 400 mg/L biotin, 20 g/L glucose, 100 mg/L Zeocin) in 1000 mL baffled shake flasks at 30 °C and 230 rpm for 24 h.

#### 3.3.2. Dynamic Batch Cultivation

For dynamic batch cultivations, 3582.6 mL 2-fold concentrated basal salt medium (BSM; 26.7 mL/L 85% phosphoric acid, 1.17 g/L CaSO_4_·2H_2_O, 18.2 g/L K_2_SO_4_, 14.9 g/L MgSO_4_·7H_2_O, 4.13 g/L KOH, 0.3 mL/L Antifoam Struktol J650, 60 g/L glycerol) were sterilized in a 5 L working volume glass bioreactor (Infors, Molndal, Sweden). After sterilization, 4.35 mL PTM1 per litre medium were added (*i.e.*, 17.4 mL for 4 litres) and pH was set to 5.0 with concentrated ammonia solution. Pre-cultures were aseptically transferred to the respective vessel (10% inoculation volume) and the batch phase on glycerol was carried out at 30 °C with the stirrer fixed at 1300 rpm. Aeration with compressed dry air was set to 1 volume per volume per minute (vvm). Dissolved oxygen (dO_2_) was measured with a sterilizable VisiFerm DO 225 probe (Hamilton, ON, Canada). The pH was measured with a sterilizable electrode (Mettler Toledo, Greifensee, Switzerland) and maintained constant at pH 5.0. Reactor weight was continuously recorded by a precision balance (Sartorius, Göttingen, Germany). Batch and methanol adaptation were performed at 30 °C. After the complete consumption of glycerol, indicated by an increase of dO_2_ and a drop in off-gas activity, the first methanol pulse at a final concentration of 0.5% volume per volume (*v*/*v*) was conducted with methanol supplemented with 12 mL/L PTM1. Following pulses were performed with 1% methanol/PTM1 (*v*/*v*) at different temperatures. At 30, 25 and 20 °C at least two pulses were applied, respectively. For each pulse, two samples were taken to determine the concentrations of substrate and product, as well as dry cell weight to calculate specific rates and yields.

#### 3.3.3. Analysis of Growth and Expression Parameters

Dry cell weight (DCW) was determined by centrifugation of 2 × 5 mL fermentation broth (4800 rpm, 10 min, 4 °C), washing the pellet with 5 mL water and subsequent drying to a constant weight at 105 °C. Optical density at 600 nm (OD_600_) was determined in a spectrophotometer (Thermo Scientific, Waltham, MA, USA). Activity of HRP in the cell-free supernatant was determined using a previously described assay in a CuBiAn-XC enzymatic robot (Innovatis, Bielefeld, Germany) [27]. Protein concentration was determined using a Bradford protein assay kit (Thermo Scientific). All growth and protein expression parameters were determined in duplicates.

#### 3.3.4. Analysis of Substrate Concentration

Concentrations of methanol were determined in cell free samples by HPLC (Agilent Technologies, Santa Clara, CA, USA) equipped with an ion-exchange column (Supelcogel C-610H, Sigma-Aldrich, St. Louis, MO, USA) and a refractive index detector (Agilent Technologies). The mobile phase was 0.1% H_3_PO_4_ with a constant flow rate of 0.5 mL/min and the system was run isocratic at 30 °C. Calibration was done by measuring standard points in the range from 0.1 to 10 g/L methanol. Measurements of biomass, product and substrate concentration were executed in duplicates.

#### 3.3.5. Calculation of Strain Physiological Parameters

The relevant parameters to physiologically characterize the recombinant yeast strains were: carbon dioxide evolution rate (CER; mmol/L/h), adaptation time to methanol (h), specific methanol uptake rate (q_MeOH_; mmol/g/h), biomass yield (Y_X/MeOH_; C-mol/C-mol), carbon dioxide yield (Y_CO2/MeOH_; C-mol/C-mol) and C-balance. Details concerning the calculation of these parameters have been published before [20,21,27].

### 3.4. Protein Purification

Cell-free cultivation broth was diafiltrated with a Centramate 500S TFF system (Pall) using a 10 kDa MWCO membrane. The buffer was HCIC-A (20 mM NaOAc, 1.0 M NaCl, pH 6.0) and the protein solution was concentrated to a final volume of 100–120 mL. The HCIC resin MEP HyperCel™ was obtained from Pall (Port Washington, NY, USA) and HCIC was performed in flow-through mode [8,19]: a column containing 25 mL of MEP HyperCel™ resin was equilibrated with at least four column volumes (CV) buffer HCIC-A. The HRP solution in HCIC-A was loaded onto the column that was then washed with at least five CV of HCIC-A at a flow rate of 20 cm/h. Then a step elution to 100% buffer HCIC-B (50 mM Tris, pH 8.0) was performed. After elution, the column was washed with five CV 0.8 M NaOH before it was stored in EtOH 20%, 1.0 M NaCl. During all the steps, fractions were collected and analyzed for protein content and catalytic activity. Fractions showing HRP activity were pooled and concentrated to around 1.5 mL using Amicon Ultra-15 Centrifugal Filter Units with 10 kDa MWCO (Merck Millipore, Darmstadt, Germany).

The efficiency of the purification was evaluated by determining the purification factor (PF; Equation (1)) and the recovery yield of HRP activity in percentage (R%; Equation (2)). The suffixes “pre” and “post” indicate the respective values before and after the HCIC step.

(1) $$\text{PF = }\frac{\text{specific activit}{\text{y}}_{\text{post}}}{\text{specific activit}{\text{y}}_{\text{pre}}}$$

(2) $$\text{R\% = }\frac{\text{volumetric activit}y_{\mathrm{post}}*\text{volum}e_{\mathrm{post}}}{\text{volumetric activit}y_{\mathrm{pre}}*\text{volum}e_{\mathrm{pre}}}$$

Additionally, Reinheitszahl values (RZ; A_404_/A_280_) of the concentrated enzyme preparations were measured. Absorbance at 280 and 404 nm were determined in a quartz cuvette in a spectrophotometer (UV-1601; Shimadzu, Long Beach, CA, USA).

### 3.5. SDS-PAGE

Apparent sizes and purities of produced HRP variants were followed by SDS-PAGE according to the Laemmli protocol [28]. Electrophoresis was done using an Amersham ECL Gel 8%–16% gel (GE Healthcare, Buckinghamshire, UK) in 1× Tris-glycine buffer. Before loading, the gel had to be pre-run at 160 V for 12 min. Protein separation was performed at 140 V for about 2 h. BLUeye Prestained Protein Ladder (GeneDirex, Taoyuan County, Taiwan) was used as protein mass standard. Gels were stained with Coomassie Blue sensitive stain.

### 3.6. Protein Identification and Peptide Analysis Using LC-ESI-MS

The relevant protein bands were cut out from the SDS gel and digested in gel. S-alkylation with iodoacetamide and digestion with sequencing grade modified trypsin (Promega, Madison, WI, USA) were performed. The peptide mixture was analysed using a Dionex Ultimate 3000 system directly linked to an ion trap instrument (amaZon speed ETD, Bruker, Billerica, NA, USA) equipped with the standard ESI source in the positive ion, DDA mode (=switching to MSMS mode for eluting peaks). MS-scans were recorded (range: 400–1600 *m*/*z*; icc target: 100,000; max. accu time: 200 ms) and the 12 highest peaks were selected for fragmentation. Instrument calibration was performed using ESIcalibration mixture (Agilent). For separation of the peptides a Thermo BioBasic C18 separation column (5 μm particle size, 150 × 0.360 mm) was used. A gradient from 97% solvent A and 3% solvent B (Solvent A: 65 mM ammonium formiate buffer, B: 100% ACCN) to 32% B in 45 min was applied, followed by a 15 min gradient from 32% B to 75% B, at a flow rate of 6 μL/min. The analysis files were converted using Data Analysis 4.0 (Bruker) to XML files, which are suitable to perform MS/MS ion searches with MASCOT (embedded in ProteinScape 3.0, Bruker) for protein identification. Only proteins identified with at least 2 peptides with a protein score higher than 80 were accepted. For the searches the SwissProt database was used. Peptide MS/MS data were evaluated against the target sequence using X! Tandem (www.thegpm.org/tandem/) with the following settings: reversed sequences no; check parent ions for charges 1, 2 and 3 yes; models found with peptide log_e_ lower −1 and proteins log_e_ lower −1; residue modifications: oxidation M, W and deamidation N, Q; isotope error was considered; fragment type was set to monoisotopic; refinement was used with standard parameters; fragment mass error of 0.3 Da and ± 50 ppm parent mass error; fragment types b and y ions; maximum parent ion charge of 3; missed cleavage sites allowed was set to 1; semi-cleavage yes.

### 3.7. Biochemical Enzyme Characterization

We determined the basic kinetic parameters *K*_m_ and *v*_max_ for the substrates ABTS and H_2_O_2_ for the different HRP variants in a spectrophotometer UV-1601 (Shimadzu). The reaction mixture with a final volume of 1.0 mL contained 20 μL of HRP variant, 50 mM potassium phosphate buffer, pH 6.5, and either varying concentrations of ABTS (0.01–10 mM) and a saturating concentration of H_2_O_2_ of 1.0 mM or varying concentrations of H_2_O_2_ (0.001–1.0 mM) and a saturating concentration of ABTS of 10.0 mM, respectively. The increase in absorption was followed at 420 nm at 30 °C for 180 s. Absorption curves were recorded with a software program (UVPC Optional Kinetics software; Shimadzu). The maximum reaction rate (*v*_max_) and the Michaelis constant (*K*_m_) were calculated with the software Sigma Plot (Systat Software Inc., Chicago, IL, USA).

The thermal stability of individual HRP variants was tested at 60 °C. The residual activity with ABTS was measured after 1, 5, 10, 15, 30, 45, 60, 90 and 120 min of incubation at 60 °C in a PCR thermoblock. Protein concentrations were normalized to 0.1 mg/mL in Bis-Tris buffer (50 mM Bis-Tris, pH 6.5) to guarantee comparability. Residual activities were plotted *versus* the incubation time and the half-life times of thermal inactivation at 60 °C (*τ*½) were calculated using Equation (3):

(3) $$\tau_{1/2}=\frac{\ln2}{\text{kin}}$$

k_in_: rate of inactivation (slope of the logarithmic residual activity).

## 4. Conclusions

In this study we combined protein and strain engineering to obtain an active and stable recombinant HRP variant with reduced surface glycosylation. We combined four mutations, which individually had shown beneficial effects on either catalytic activity or thermal stability before, and expressed this enzyme variant as well as the unmutated wildtype enzyme in both an unmodified *P. pastoris* benchmark strain and a Δoch1 strain. Our results can be summarized as:

- Production of the 4/8 HRP variant caused a physiological burden for the *P. pastoris* benchmark strain slowing down its metabolism. In contrast, production of the same enzyme variant did not affect physiology of the Δoch1 strain. While q_s MeOH_ increased with increasing temperature for the benchmark strain, the opposite was true for the Δoch1 strain. Based on strain physiological parameters identified in dynamic batch experiments, fed-batch strategies can be easily designed for future production of these enzyme variants.
- Reduced and missing surface glycosylation did not affect substrate affinity of HRP, but significantly reduced catalytic activity.
- Introducing the four mutations into HRP significantly boosted thermal stability. In fact, the 4/8 HRP variant produced in the *P. pastoris* benchmark strain even showed a 3.3-fold increased thermal half-life time compared to the HRP preparation from plant.
- Considering both, enzyme activity and thermal stability, the 4/8 HRP variant produced in the *P. pastoris* benchmark strain might be interesting for future applications in medical diagnostics. This enzyme variant can be efficiently produced in the bioreactor, shows considerable catalytic activity and high thermal stability and is less glycosylated, which might allow more controlled and efficient conjugation to antibodies and lectins.

In this study we show that enzymes can be modified not only by mutation, but that a combination of both strain and protein engineering is a useful way to obtain enzyme variants tailored to specific needs.

## Supplementary Materials

Supplementary materials can be found at http://www.mdpi.com/1422-0067/16/10/23127/s1.
