# Supplementary material for: Combining Protein and Strain Engineering for the Production of Glyco-Engineered Horseradish Peroxidase C1A in Pichia pastoris
Source: Int J Mol Sci. 2015 Sep 24;16(10):23127–42. doi: 10.3390/ijms161023127 (PMC4632689; doi:10.3390/ijms161023127)
Supplement: Supplementary file 1 [file ijms-16-23127-s001.pdf]

Supplementary Information

Table S1. Oligonucleotide primers to mutate the 4 Asn residues N13, N57, N255 and N268 of the enzyme HRP C1A, which act as *N*-glycosylation sites, to either Asp or Ser. The mutation sites are depicted in italics.

N-Site	Name	Sequence (5′→3′)
N13	N13D_fwd	AAC TCT TGT CCT *GAT* GTG TCC AAC ATC
	N13_rev	AGG ACA AGA GTT ATC GTA GAA GGT TGG AGT
N57	N57S_fwd	TCC ATC TTG CTG GAC *AGC* ACT ACC TC
	N57_rev	GTC CAG CAA GAT GGA AGC ATC ACA ACC
N255	N255D_fwd	TTG TTC TCC TCT CCT *GAC* GCT ACT GAT
	N255_rev	AGG AGA GGA GAA CAA CTC CTG GTC
N268	N268D_fwd	G AGA TCC TTC GCA *GAC* TCC ACT CAA
	N268_rev	TGC GAA GGA TCT CAC CAA TGG AAT G
